# Emergence and transmission of the high-risk ST78 clone of OXA-48-producing *Enterobacter hormaechei* in a single hospital in Taiwan

**DOI:** 10.1080/22221751.2024.2404165

**Published:** 2024-09-11

**Authors:** Chih-Ming Chen, Hui-Ling Tang, Ying-Tsong Chen, Se-Chin Ke, Yi-Pei Lin, Bo-Han Chen, Ru-Hsiou Teng, Chien-Shun Chiou, Min-Chi Lu, Yi-Chyi Lai

**Affiliations:** a Department of Internal Medicine, Tungs’ Taichung MetroHarbor Hospital, Taichung, Taiwan; b College of Medicine, National Chung Hsing University, Taichung, Taiwan; c Department of Microbiology and Immunology, School of Medicine, China Medical University, Taichung, Taiwan; d Institute of Genomics and Bioinformatics, National Chung Hsing University, Taichung, Taiwan; e Infection Control Office, Tungs’ Taichung MetroHarbor Hospital, Taichung, Taiwan; f Department of Medical Technology, Jen-The Junior College of Medicine, Nursing and Management, Miaoli, Taiwan; g Department of Medical Research, Tungs’ Taichung MetroHarbor Hospital, Taichung, Taiwan; h Central Region Laboratory, Center for Diagnostics and Vaccine Development, Centers for Disease Control, Ministry of Health and Welfare, Taipei, Taiwan; i Division of Infectious Diseases, Department of Internal Medicine, China Medical University Hospital, Taichung, Taiwan; j Department of Microbiology and Immunology, School of Medicine, Chung Shan Medical University, Taichung, Taiwan; k Department of Internal Medicine, Chung Shan Medical University Hospital, Taichung, Taiwan

**Keywords:** *Enterobacter hormaechei*, Carbapenemase genes, ST78, OXA-48, *mcr-9*.*1*, resistome, plasmidome

## Abstract

Carbapenem-resistant *Enterobacter cloacae* complex is a significant global healthcare threat, particularly carbapenemase-producing *Enterobacter hormaechei* (CPEH). From January 2017 to January 2021, twenty-two CPEH isolates from a regional teaching hospital in central Taiwan were identified with the carriage of carbapenemase genes *bla*_KPC-2_, *bla*_IMP-8_*,* and predominantly *bla*_OXA-48_. Over 80% of these CPEH strains clustered into the high-risk ST78 lineage, carrying a *bla*_OXA-48_ IncL plasmid (pOXA48-CREH), nearly identical to the endemic plasmid pOXA48-KP in ST11 *Klebsiella pneumoniae*. This OXA-48-producing ST78 lineage disseminated clonally from 2018 to 2021 and transferred pOXA48-CREH to ST66 and ST90 *E. hormaechei*. An IMP-8-producing ST78 strain harbouring a *bla*_IMP-8_-carrying pIncHI2 plasmid appeared in 2018, and by late 2020, a KPC-2-producing ST78 strain was identified after acquiring a novel *bla*_KPC-2_-carrying IncFII plasmid. These findings suggest that the high-risk ST78 lineage of *E*. *hormaechei* has emerged as the primary driver behind the transmission of CPEH*.* ST78 has not only acquired various carbapenemase-gene-carrying plasmids but has also facilitated the transfer of pOXA48-CREH to other lineages. Continuous genomic surveillance and targeted interventions are urgently needed to control the spread of emerging CPEH clones in hospital settings.

## GRAPHICAL ABSTRACT

**Figure:**
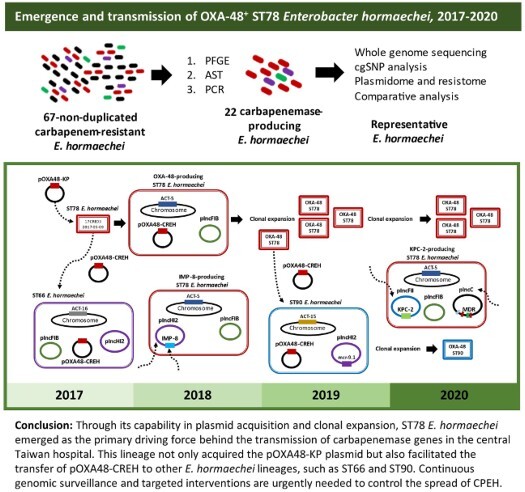

## Introduction

Carbapenem-resistant *Enterobacterales* has emerged as a significant global healthcare threat over the past decade, presenting challenges in treating infections due to limited treatment options [1]. Among the top five nosocomial species of *Enterobacterales* causing bloodstream infections, the *Enterobacter cloacae* complex (ECC) stands out prominently. Within ECC, *Enterobacter hormaechei* emerges as a predominant pathogen associated with various infections [2]. An overall genome-related index suggested that *E. hormaechei* contains at least five subspecies, including *oharae*, *steigerwaltii*, *hormaechei*, *hoffmannii*, and *xiangfangensis* [3]. Characterized by its ability to acquire multiple antimicrobial resistance (AMR) genes through horizontal gene transfer from other *Enterobacterales*, *E. hormaechei* has increasingly been linked to nosocomial outbreaks [4–6].

Notably, carbapenemase-producing *E. hormaechei* (CPEH), including those producing KPC, NDM, GIM, and IMP enzymes, have been documented in various studies [4, 6–8]. The OXA-48 carbapenemase was initially identified in a carbapenem-resistant *Klebsiella pneumoniae* strain in Turkey in 2001 and arrived in Taiwan at the end of 2013 [9]. Through acquiring a variant of the epidemic *bla*_OXA-48_-carrying IncL plasmid, pOXA-48, ST11_KL64 *K. pneumoniae* emerged as one of the primary clones of carbapenemase-producing *K. pneumoniae*, which peaked during 2013-2015 [10] and has shown a constantly increasing prevalence in hospitals in Taiwan [11]. Inter-species transfer of pOXA-48 has been suggested as a contributing factor to the emergence and spread of OXA-48-producing *E. hormaechei* among companion animals and humans, as evidenced by a recent study in Swiss hospitals [12]. Through the acquisition of *bla*_OXA-48_-carrying IncL plasmids, high-risk international clones ST66, ST171, and ST78 of ECC have emerged as the etiological agents of bacteremia in a Spanish hospital [13].

In May 2017, we identified the first OXA-48-producing *E. hormaechei* isolate from a patient with bacteremia in our hospital. In addition to *bla*_OXA-48_, we isolated several carbapenemase-producing *E. hormaechei*, including KPC-2- and IMP-8-producing isolates. By January 2021, 22 carbapenemase-producing *E. hormaechei* (CPEH) were identified out of 67 non-duplicated carbapenem-resistant isolates. To elucidate the molecular mechanisms contributing to the emergence and dissemination of CPEH in our hospital, we utilized conventional profiling methods and next-generation whole genome sequencing techniques, including Illumina and MinION sequencing. This comprehensive approach enabled us to characterize the strains and examine their possible phylogenetic relatedness, shedding light on the dynamics of AMR gene acquisition and spread for this clinically significant pathogen.

## Materials and methods

***E. hormaechei* isolates.** Positive cultures indicating carbapenem-resistant *E. hormaechei* were collected from patients admitted to Tung’s Taichung Metro Harbour Hospital, a regional teaching hospital with 24 clinical departments and 1,381 beds. Between January 2017 and January 2021, 67 non-duplicate isolates associated with either colonization or infections were included in this study. Species identification was conducted using the Bruker MALDI Biotyper™ and confirmed via whole genome sequencing, which was based on an average nucleotide identity (ANI) of over 96%, using *E. hormaechei* (CP017186) as the type strain for species delineation. Antimicrobial susceptibility testing was performed using the Phoenix Automatic Microbiology System (BD Diagnostics, MD, USA), with interpretations based on CLSI breakpoints (M100-S27). The minimal inhibitory concentration of colistin was determined using the broth dilution method**.**

**Pulse-filed gel electrophoresis (PFGE), multi-locus sequence typing (MLST), and detection of carbapenemase genes, *mcr-9*, and the replicon region of IncL and IncHI2 plasmid**. Following a standardized protocol for the subtyping of *Enterobacteriaceae* [14], pulse-filed gel electrophoresis (PFGE) was performed with the CHEF-DR III system (Bio-Rad Laboratories Inc, USA) to determine the clonal relatedness of carbapenem-resistant *E. hormaechei* (n = 67). The profiles of the *Xba*I macro-restricted fragments of each isolate were analyzed with the Dice coefficients and the unweighted pair group method with an arithmetic mean algorithm with a 1.5% optimization value and 1.5% position tolerance using the analytic tools provided by the GelCompar II 6.5 software (Applied Maths, Belgium). *Xba*I-digested DNA samples from *Salmonella enterica* subsp. *enterica* serotype Braenderup H9812 was used as molecular size markers. Clusters of PFGE-*Xba*I were determined in the dendrogram using a 75% similarity cut-off. The sequence types (STs) of the representative strains were determined using the MLST scheme established for *E. cloacae* [15]. This scheme is based on the sequences of seven housekeeping genes: *aspC*, *clpX*, *fadD*, *icd*, *mdh*, *recA*, and *purA* (http://pubmlst.org/ecloacae). Additionally, *bla*_ACT_ was amplified using polymerase chain reaction (PCR) with specific primers (supplement Table S1) and subjected to Sanger sequencing for genotyping. Genomic DNA extracted from each carbapenem-resistant *E. hormaechei* (CREH; n = 67) was subjected to the detection of carbapenemase-coding genes, including *bla*_KPC_, *bla*_OXA-48_, *bla*_IMP_, *bla*_NDM_, and *bla*_VIM_, and *mcr-9* gene by PCR with specific primers as previously described [16, 17]. The carriage of IncL-type and IncHI2-type plasmids was detected using PCR with specific primers targeting the IncL and IncHI2 replicons, respectively (supplement Table S1).

**Whole genome sequencing.** The genomic DNA of representative carbapenemase-producing *E. hormaechei* isolates was extracted and subjected to whole genome sequencing by Illumina MiSeq sequencer (Illumina, San Diego, USA) and Nanopore MinION sequencer (Oxford Nanopore Technologies, Oxford, UK), according to the standard protocol provided by the manufacturer, respectively. Qualified reads yielded from Illumina and MinION were assembled with Unicycler *v.*0.4.8. The final assembly was polished by Unicycler using Illumina reads, and the rate of minor base-level errors was reduced by Pilon. Assemblies with a size ≤ 1000 kb containing plasmid replicons were extracted from the assembly graph with BANDAGE *v.*0.8.1. The genome assemblies of *E. hormaechei* strains in this study, which can be downloaded through the link: https://reurl.cc/34aboX, are publicly available in GenBank under BioProject PRJNA791797.

**Genome profiling and comparative genomic analysis.** Genome assemblies were annotated by RAST (https://rast.nmpdr.org/) and manually curated. Antimicrobial genes were identified with ResFinder, and plasmid incompatibility (Inc) groups were assessed with PlasmidFinder from the Centre for Genomic Epidemiology (http://www.genomicepidemiology.org/). Comparative sequence alignments were performed with Geneious Prime 2022.1.1 (Biomatters, New Zealand). Alignment and visualization of plasmids were performed with BLAST Ring Image Generator (BRIG v.0.95) [18]. Mauve alignment and comparative genomics analysis were performed with Geneious Prime.

**cgSNP analysis.** Parsnp 1.2, with default parameters [19], was utilized for core genome alignment, SNP calling, and phylogenetic tree construction in the genome assemblies of carbapenemase-producing *E. hormaechei*, using ST78 *E. hormaechei* subsp. *hoffmannii* strain Eh1 (GCA_009834325.1) as the reference. The resulting cgSNP phylogeny tree was visualized in Geneious Prime, with the corresponding metadata, including isolation time, ST, type of plasmids, and the carriage of AMR genes.

## Results

**Figure 1.**
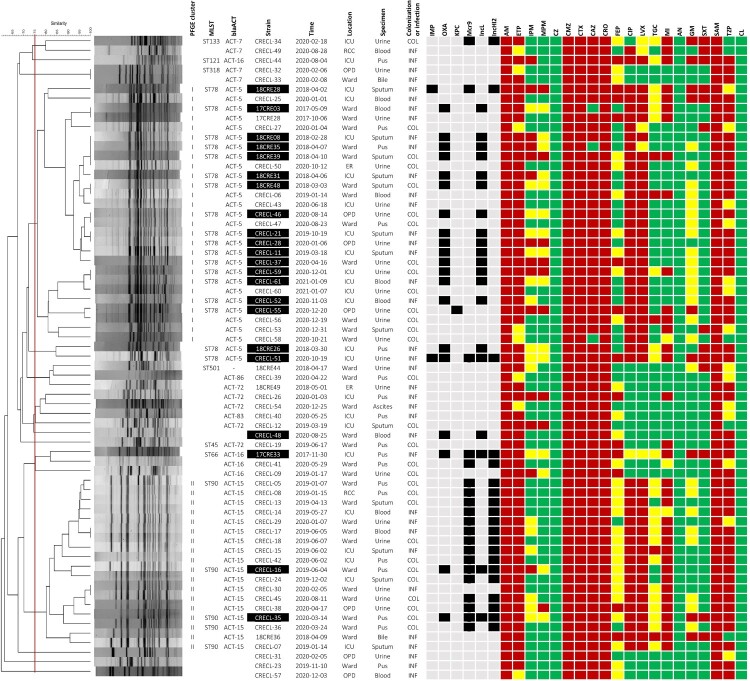
Carbapenem-resistant *E. hormaechei* (CREH) isolates (n = 67) collected from January 2017 to January 2021 were clustered based on PFGE-*Xba*I profiles. For each non-duplicated isolate, features such as isolation time, location, specimen type, and colonization or infection with CREH are presented. In addition to antibiotic susceptibility testing results, the presence of specific carbapenemase genes (*bla*_OXA_, *bla*_KPC_, *bla*_IMP_) or the *mcr-9.1* gene, as well as the carriage of IncL- and IncHI2-type plasmid replicons, was detected by PCR and is indicated in black. The absence of these genetic loci is shown in light grey. Sequence types (STs) were determined by the MLST scheme for *E. cloacae* (http://pubmlst.org/ecloacae). Genotyping of *bla*_ACT_ was performed by sequencing PCR products amplified with specific primers (Table S1). Strains with one of the carbapenemase genes (*bla*_OXA_, *bla*_KPC_, *bla*_IMP_) were classified as CPEH (n = 22) and highlighted with white text on a black background. Colour coding indicates antibiotic resistance: red for resistant, yellow for intermediate, and green for susceptible. Abbreviations for antibiotics are as follows: AN (amikacin), GM (gentamycin), AM (ampicillin), SAM (Ampicillin-Sulbactam), CZ (cefazolin), CMZ (cefmetazole), CTX (cefotaxime), CAZ (ceftazidime), CRO (ceftriaxone), FEP (cefepime), TZP (Piperacillin-Tazobactam), ETP (ertapenem), IMP (imipenem), MPM (meropenem), CL (colistin), CIP (ciprofloxacin), LVX (levofloxacin), TGC (tigecycline), MI (minocycline), SXT (Trimethoprim-Sulfamethoxazole).

**Figure 2.**
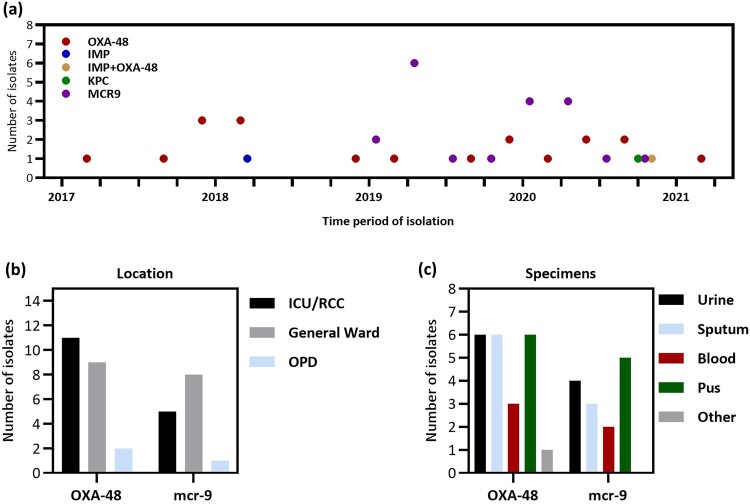
**Emergence of carbapenemase-producing *E. hormaechei* (CPEH). (a)** The time scale of clinical isolates of *E. hormaechei* carrying *bla*_IMP_ (blue), *bla*_OXA-48_ (red), *bla*_KPC_ (green), and *mcr-9.1* (purple) gene, detected by PCR. **(b)** The locations of patients with *bla*_OXA-48_ or *mcr-9.1*-positive *E. hormaechei* colonization or infections are presented, including the intensive care unit (ICU), respiratory care centre (RCC), general ward, and outpatient department (OPD). **(c)** The type of specimens from which *bla*_OXA-48_ or *mcr-9.1*-positive *E. hormaechei* were isolated included urine, sputum, blood, pus, and others.

**Emergence of carbapenemase-producing *E. hormaechei*.** Over a span of four years, we isolated 67 nonduplicate carbapenem-resistant *E. hormaechei* (CREH; Figure 1). A significant finding occurred in May 2017 when we first isolated a carbapenemase-producing *E. hormaechei* (CPEH) carrying the *bla*_OXA-48_ gene from a patient with bacteremia. Subsequently, OXA-48-producing *E. hormaechei* strains were consistently found in various specimens associated with colonization or infections. Additionally, starting in 2019, we observed the co-occurrence of the *mcr9* gene with *bla*_OXA-48_ in several isolates. By January 2021, out of the 67 CREH isolates, 22 were identified as carbapenemase-producing *E. hormaechei* (CPEH). These included one KPC-positive, one IMP-positive, one IMP-OXA-48-positive, and 19 OXA-48-positive strains (Figure 2a). The intensive care unit (ICU) was the location where OXA48- and *mcr9*-positive *E. hormaechei* isolates were most frequently encountered. Moreover, most OXA-48-positive *E. hormaechei* were isolated from urine, sputum, or pus, while *mcr9*-positive isolates were predominantly collected from pus specimens (Figure 2b).

**Figure 3.**
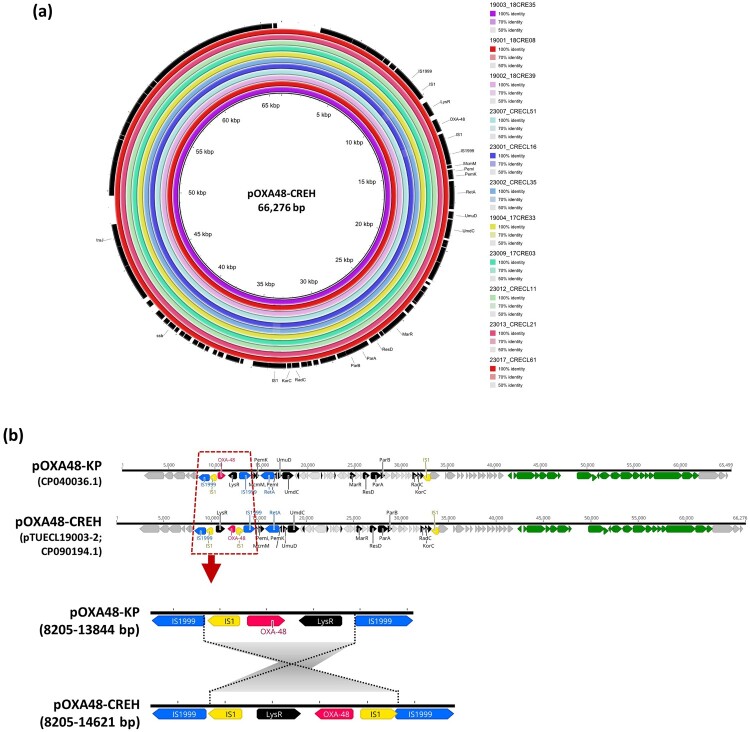
**Acquisition of an IncL-type pOXA48 plasmid in *E. hormaechei* Isolates. (a)** BRIG comparison of the *bla*_OXA-48_-carrying IncL plasmid, named pOXA48-CREH, in 11 clinical isolates collected in this study, aligned against pOXA48-CREH of 18CRE35 (CP090194.1). **(b)** Alignment of pOXA48-CREH with the endemic plasmid pOXA48-KP (CP040036.1) found in ST11_KL64 *K. pneumoniae*. The grey-shaded connection indicates an inversion between two copies of IS*1999*, causing IS*1* (yellow) duplication on pOXA48-CREH.

**Acquisition of the endemic *bla*_OXA-48_-carrying IncL plasmid by *E. hormaechei* ST78, ST66, and ST90.** Based on the cut-off of 75% similarity of PFGE-*Xba*I pulsotypes, the majority of the 67 CREH isolates were grouped into two major clusters, I and II (Figure 1). The sequence types correlated to the PFGE cluster I and II were ST78 and ST90, respectively. Of 22 CPEH strains, 18 belonged to ST78, and 2 were in the ST90 cluster. The remaining two CPEH isolates were 17CRE33, classified as ST66 and CRECL48, for which the ST could not be determined due to poor sequence quality. Regardless of the sequence type, all the OXA-48-positive *E. hormaechei* carried a *bla*_OXA-48_-carrying IncL plasmid, designated as pOXA48-CREH (Figure 3a). This plasmid, sized at 66,276 bp, exhibited close relatedness to the endemic *bla*_OXA-48_-carrying IncL plasmid frequently carried by ST11 *K. pneumoniae* in Taiwan. Like OXA-48 *K. pneumoniae*, the IncL plasmid harboured *bla*_OXA-48_ within a Tn*1999.2* transposon in *E. hormaechei*. An inversion occurred between two copies of IS*1999*, leading to a duplication of IS*1* on pOXA48-CREH (Figure 3b).

**Figure 4.**
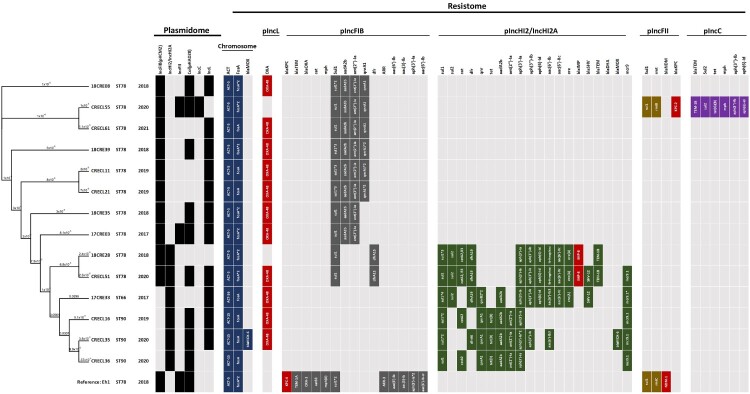
**Phylogenomic relatedness, plasmidome, and resistome of representative carbapenemase-producing *E. hormaechei* strains.** A phylogenetic tree was constructed based on cgSNP analysis of representative *E. hormaechei* strains (n = 14) using *E. hormaechei* Eh1 (GCA_009834325.1) as the reference. The distance between nodes is presented as the substitution rate per site. The plasmid Inc-type and the carriage of antimicrobial resistance (AMR) genes were determined by PlasmidFinder and ResFinder from the Centre for Genomic Epidemiology (http://www.genomicepidemiology.org/). The absence of the indicated AMR gene is shown in light grey.

**Figure 5.**
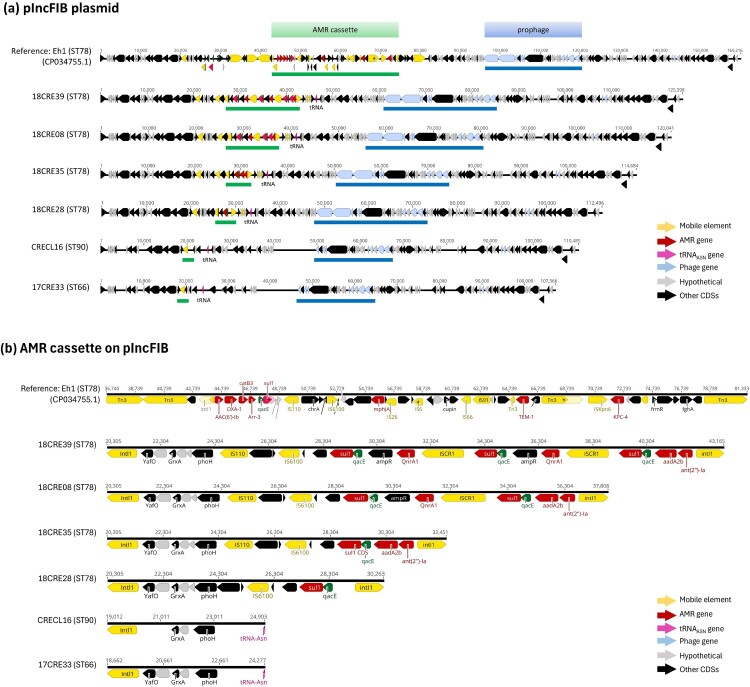
**Comparison of pIncFIB-CREH**. **(a)** Alignment of pIncFIB plasmids in representative *E. hormaechei* ST78, ST90, and ST66 strains. **(b)** Alignment of the class I integron-flanked AMR cassettes on the pIncFIB plasmids identified in representative strains in this study against the Tn*3*-flanked AMR cassette on the pIncFIB plasmid (CP034755.1) of the ST78 reference strain Eh1.

**Figure 6.**
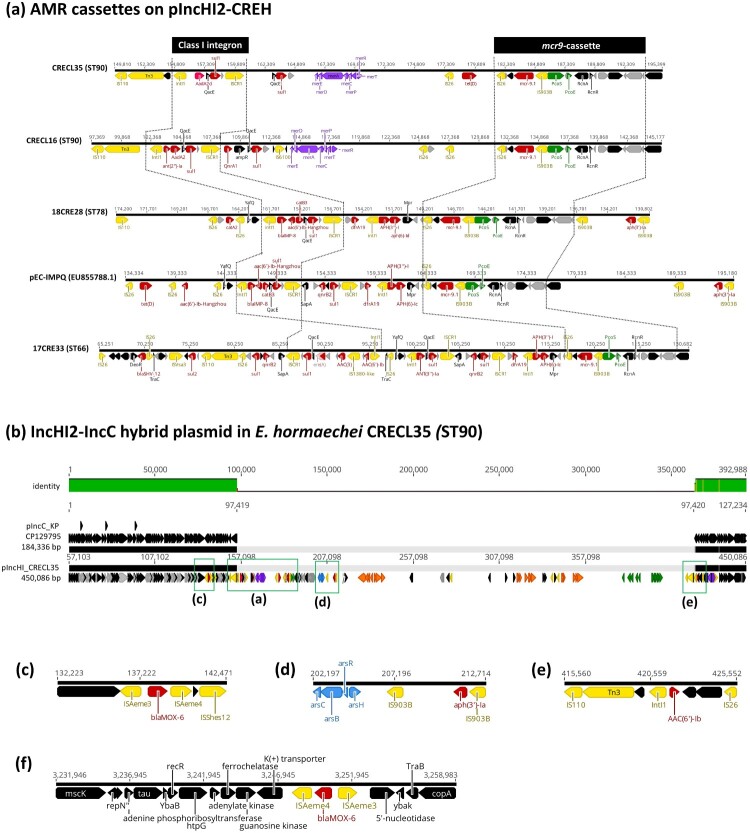
**Comparison of AMR cassettes on pIncHI2-CREH. (a)** Alignment of the region containing major AMR cassettes of the pIncHI2 plasmids in representative *E. hormaechei* ST90, ST78, and ST66 strains and the corresponding region of the closely related plasmid pEC-IMPQ (EU855788.1). **(b)** Alignment of the large IncHI2-IncC hybrid plasmid (450,336 bp) identified in CRECL35 (ST90) against the IncC plasmid (CP129795.1; 184,336 bp) in *K. pneumoniae* 2020C07-229 (SAMN36281330). **(c-e)** Detailed presentation of other AMR cassettes on the pIncHI2-IncC hybrid plasmid in CRECL35. **(f)** Insertion of *bla*_MOX-6_ into the chromosome of CRECL35 (ST90).

**Phylogenomic relatedness, plasmidome, and core resistome of carbapenemase-producing *E. hormaechei*.** To further investigate the relationships among CPEH isolates, we selected 14 representative strains for whole genome sequencing analysis, which included 10 CPEH strains of ST78, 3 strains of ST90, and one strain of ST66. Genome assemblies of the 14 strains were subjected to cgSNP analysis using ST78 *E. hormaechei* subsp. *hoffmannii* strain Eh1 as the reference (Figure 4). All *E. hormaechei* harboured the glutathione S-transferase gene *fosA* and the AmpC β-lactamase gene *bla*_ACT_ within their chromosomes, conferring resistance to fosfomycin and cephalosporins. The subtype of *bla*_ACT_ correlated with the sequence type, with *bla*_ACT-5_ associated with ST78 and *bla*_ACT-15_ with ST90 (supplement Figure S1). The predominant drug-resistant plasmid carried by ST78 *E. hormaechei* was an IncFIB-type plasmid, ranging in size from 110 to 160 kb (Figure 5a). Unlike the Tn*3*-mediated acquisition of a *bla*_KPC-4_-containing antimicrobial resistance (AMR) cassette in the reference strain *E. hormaechei* Eh1, *E. hormaechei* isolates in this study acquired AMR cassettes through class I integron-mediated transfer (Figure 5b). In contrast to ST78, the class I integron region on the pIncFIB plasmid carried by ST66 or ST90 isolates did not contain AMR genes. Instead, ST90 *E. hormaechei* harboured most AMR genes on a large IncHI2/HI2A-type plasmid (named pIncHI2_CREH), ranging in size from 264 to 450 kb, which was also harboured by some ST78 and ST66 strains (Figure 4). The pIncHI2_CREH plasmids were closely related to pEC-IMPQ (EU855788.1) (supplement Figure S2), whose variants were frequently detected in clinical isolates of *E. cloacae* complex [20]. Most pIncHI2_CREH plasmids (5/7) carried a conserved *mcr-9* genetic context as IS*26*-*wbuC*-*mcr-9.1*-IS*903B*-*pcoS*-*pcoE*-*rcnA*-*rcnR* [21]. Additionally, ST78 strains concurrently acquired the metallo-lactamase gene, *bla*_IMP-8_, on their pIncHI2 plasmid (Figure 6a). The largest pIncHI plasmid was detected in the ST90 *E. hormaechei* strain CRECL35 (450,086 bp), which was a hybrid of pIncHI2_CREH and an IncC-type plasmid identical to that (CP129795.1) found in a *K. pneumoniae* isolate (SAMN36281330) in Taiwan (Figure 6b). Besides the class I integron-mediated acquisition of AMR cassette (Figure 6a), pIncHI2_CRECL35 also harboured several AMR genes through other insertion sequences, such as IS*Aeme*-*bla*_MOX-6_, IS*903B*-*aph(3″)-Ia*, and Tn*3*-*AAC(6′)-Ib* (Figure 6c-e). Furthermore, besides being carried by plasmid pIncHI2, the AmpC β-lactamase gene *bla*_MOX-6_ was also inserted into the chromosome of CRECL35 (Figure 6f).

**Figure 7.**
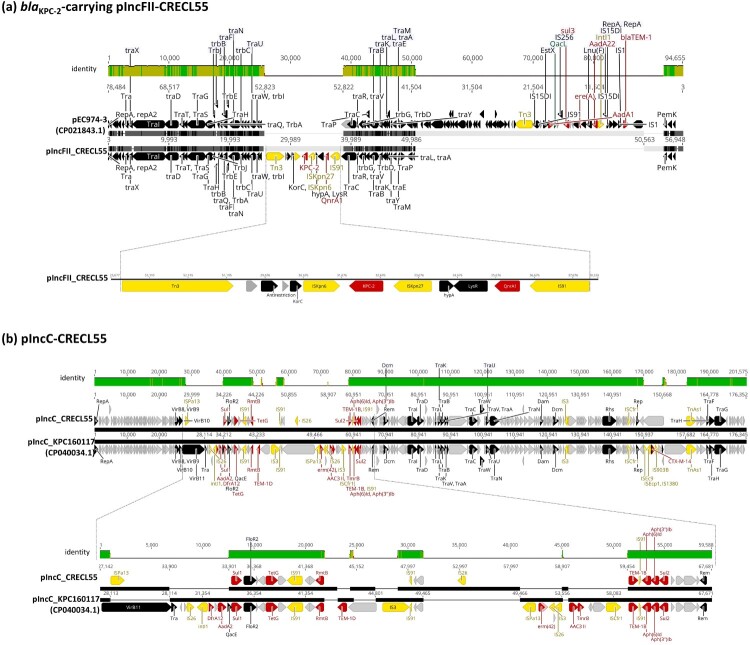
Acquisition of a ***bla***_KPC-2_-carrying pIncFII and a multi-drug-resistance pIncC plasmid in ST78 ***E. hormaechei*** strain CRECL55. (a) Mauve alignment of the pIncFII plasmid in *E. hormaechei* CRECL55 (ST78) with pEC974-3 (CP021843.1) identified in *E. coli* strain EC974 (SAMN07192703). **(b)** Mauve alignment of the pIncC plasmid in CRECL55 with the closely-related plasmid pIncC-L117 (CP040034.1) identified in *K. pneumoniae* KPC160117 (SAMN11246288).

**Acquisition of a novel *bla*_KPC-2_-carrying pIncFII plasmid and a multi-drug-resistance IncC-type plasmid by an *E. hormaechei* ST78 strain.** Towards the end of 2020, we isolated a *bla*_OXA-48_-negative but *bla*_KPC-2_-positive *E. hormaechei* strain (CRECL55). Unlike the other ST78 strain CRECL51, which carried *bla*_IMP-8_ on the large pIncHI plasmid, CRECL55 acquired a novel pIncFII plasmid. This plasmid, pIncFII-CRECL55, shared a backbone structure with the conjugal plasmid pEC974.3 (CP021843) found in *Escherichia coli* EC974 (SAMN07192703), an isolate from a southern Taiwan hospital [22]. However, the AMR cassette content on pIncFII-CRECL55 differed from that on pEC974.3. The conserved structure of *bla*_KPC-2_ cassette, identified as IS*Kpn6*-*bla*_KPC-2_-IS*Kpn27* [23], along with *qnrA1*, was incorporated into pIncFII-CRECL55 through a Tn*3*-based mobilization (Figure 7a). In addition to the novel *bla*_KPC-2_-carrying plasmid, CRECL55 also acquired an IncC-type plasmid. This plasmid is similar to a common pIncC plasmid found in ST11 *K. pneumoniae* in Taiwan [24] but harboured fewer AMR genes (Figure 7b).

## Discussion

More than 80% of the carbapenemase-producing *E. hormaechei* isolates collected in this study were clustered into a group belonging to ST78 (Figure 1). ST78 is recognized as a high-risk international clone with a unique ability to acquire various antimicrobial resistance (AMR) gene-carrying plasmids [6]. Notable examples include the *bla*_NDM-7_-IncX3 plasmid in isolates from Spain [6] and the *bla*_IMP-1_-carrying class I integron on IncW and IncFIB plasmids in isolates from Japan [25]. The first OXA-48-producing *E. hormaechei* isolate was identified in May 2017. The *bla*_OXA-48_-carrying IncL plasmid acquired by *E. hormaechei* was nearly identical to the endemic plasmid (pOXA48-KP) frequently found in ST11_KL64 *K. pneumoniae* in Taiwan [24]. The IncL plasmid exhibits high plasmid stability and strong conjugal transfer ability *in vitro* [26] and can also be horizontally transferred between enterobacteria within the gut microbiota of colonized patients [27]. The conserved conjugal transfer region of pOXA48-CREH (supplement Figure S3a) supports a plausible scenario in which ST78 *E. hormaechei* acquired the pOXA48-KP plasmid from *K. pneumoniae* through co-colonization within patients. Subsequently, an ST66 *E. hormaechei* acquired pOXA48-CREH, potentially transferred from an OXA-48-producing ST78 strain. Nevertheless, clonal expansion of pOXA48-CREH-carrying lineages was predominantly noted in ST78 *E. hormaechei*, beginning in 2018. By 2019, pOXA48-CREH was transmitted from ST78 to ST90, another high-risk clone of *E. hormaechei,* but it disseminated on a small scale in the hospital setting.

**Figure 8.**
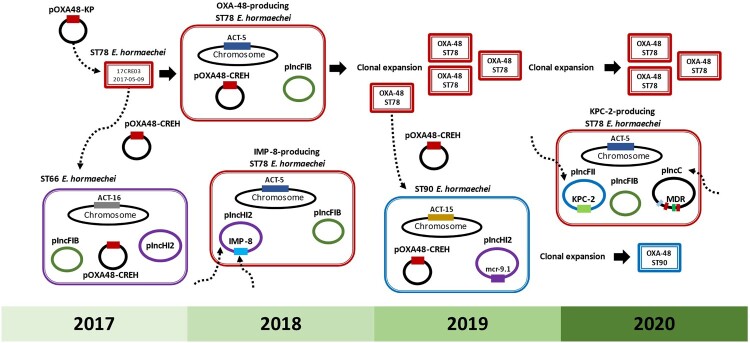
**Emergence and transmission of carbapenemase-producing *E hormaechei* during 2017-2020.** The index OXA-48-producing *E. hormaechei* strain emerged in May 2017, potentially transferring the *bla*_OXA-48_-carrying IncL plasmid, likely acquired from ST11 *K. pneumoniae*, to an ST66 *E. hormaechei* isolate. Clonal expansion of this OXA-48-producing ST78 lineage has occurred since 2018. In 2019, an ST90 *E. hormaechei* strain acquired pOXA48-CREH, probably from the OXA-48-producing ST78 lineage, and disseminated on a small scale. Through acquiring a novel *bla*_KPC-2_-carrying pIncFII plasmid and a variant of the pIncC plasmid, a KPC-2-producing ST78 *E. hormaechei* was identified at the end of 2020. Echoing the capability of ST78 *E. hormaechei* in acquiring various types of drug-resistance plasmids, in this high-risk lineage, an IMP-8-producing *E. hormaechei* was initially found in 2018 after harbouring a *bla*_IMP-8_-pIncHI2 plasmid. Collectively, ST78 *E. hormaechei* emerged as the primary driving force behind the transmission of carbapenemase genes in this hospital setting.

Despite ST66, ST78, and ST90 lineages all acquiring pOXA48-CREH, the primary lineage responsible for the clonal dissemination of carbapenemase-producing *E. hormaechei* in the regional teaching hospital was ST78 (Figure 8). The capability of ST78 *E. hormaechei* to acquire multi-drug resistance plasmids was further demonstrated by the isolation of 18CRE28 in 2018, which acquired a *bla*_IMP-8_-carrying pIncHI2 plasmid, and the KPC-2-producing strain CRECL55 in 2020. Notably, CRECL55 harboured a novel *bla*_KPC-2_-carrying pIncFII plasmid, likely originating from *E. coli* and a variant of the pIncC plasmid from ST11 *K. pneumoniae* (Figure 7). Continuous monitoring and genomic surveillance are crucial for targeted interventions to curb the spread of this high-risk clone.

Since its identification in 2019, *mcr-9.1*, a plasmid-mediated colistin resistance gene, has rapidly spread among clinical carbapenem-resistant *Enterobacterales*, primarily via IncHI2 plasmids [28]. Co-carriage of *mcr-9* and *bla*_VIM_ on pIncHI2 plasmids have been identified in *E. hormaechei* isolates in Czech Hospitals [29]. The acquisition of the *mcr-9* gene cassette between IncHI2 plasmids is thought to be mediated by an IS*903*-dependent mechanism. In our study, *mcr-9.1* was predominantly detected in ST90 *E. hormaechei* and occasionally found in ST78 and ST66 isolates. The conserved *mcr-9.1* genetic context was carried by pIncHI2 plasmids, which vary in size from 264 to 450 kb and contain a diverse array of AMR genes (Figure 6) as well as genes for conjugal transfer (supplement Figure S2a and S3b). Although the *qseBC* two-component system is required to express *mcr-9.1*, all the *mcr-9.1*-positive isolates in this study were *qseBC*-negative and consequently exhibited phenotypic susceptibility to colistin. Despite the minimal phenotypic impact of the *mcr-9.1*-carrying pIncHI2 plasmids on colistin resistance, the insertion of the *bla*_IMP-8_-containing class I integron on pIncHI2 plasmids in ST78 *E. hormaechei* (Figures 4 and 6) significantly enhances the advantage of this international high-risk clone under antimicrobial pressure. Co-carriage of *mcr-9.1*-pIncHI2 plasmid with a carbapenemase-encoding plasmid, such as pOXA48-CREH, has also been demonstrated in a clonal outbreak in a tertiary hospital in China where the majority of ST78 *E. hormaechei* co-harboured an IncFII-type plasmid encoding the class B metallo-β-lactamase NDM-1 and the pIncHI2-*mcr-9.1* plasmid [5].

Regardless of sequence type, all the representative CPEH strains had an AmpC β-lactamase gene *bla*_ACT_ in their chromosomes. The intrinsic presence of a constitutive AmpC β-lactamase gene in *Enterobacter* species has been shown to confer resistance to ampicillin, amoxicillin, and first- and second-generation cephalosporins, such as cefazolin and cefmetazole [30]. This study revealed a link between specific *bla*_ACT_ subtypes and the sequence type of *E. hormaechei*: *bla*_ACT-5_ in ST78 and *bla*_ACT-15_ in ST90 (supplement Figure S1). This linkage reflected the intrinsic inheritance of the AmpC β-lactamase gene in sub-lineages of *E. hormaechei*. All representative ST78 and ST90 CPEH strains exhibited resistance to ciprofloxacin and levofloxacin (Figure 1). However, no mutations in the quinolone resistance-determining regions (QRDRs) of *gyrA* and *parC* were identified in their chromosomes. Instead, the plasmid-borne genes *qnrA1* and *aac(6′)-Ib*-Hangzhou, which encode Qnr proteins and an aminoglycoside acetyltransferase variant, respectively, conferred resistance to fluoroquinolones in ST78 or ST90 CPEH strains (Figure 4).

From May 2017 to January 2021, 22 strains were identified as carbapenemase-producing *E. hormaechei* (CPEH), with the presence of *bla*_KPC-2_, *bla*_IMP-8_, and predominantly *bla*_OXA-48_. Notably, all OXA-48-positive strains carried a *bla*_OXA-48_-carrying IncL plasmid. Phylogenetic and comparative genomic analyses revealed the acquisition of a ∼66-kb *bla*_OXA-48_-carrying IncL plasmid by strains of different sequence types, including ST78, ST66, and ST90. Apart from duplication of IS*1*, the *bla*_OXA-48_ plasmid carried by *E. hormaechei* was nearly identical to the endemic *bla*_OXA-48_ plasmid found in ST11_KL64 *K. pneumoniae* (Figure 3). Since the end of 2013, OXA-48-producing *K. pneumoniae* has emerged and disseminated in Taiwan, including this regional teaching hospital [31]. The IncL-type *bla*_OXA-48_-carrying plasmid is highly conjugative. The pOXA48-KP-like plasmid was also identified in a recent outbreak of OXA-48-producing *Salmonella* Goldcoast from December 2020 to January 2021 in central Taiwan [32]. Furthermore, a recent study in Switzerland demonstrated a potential transfer of a ∼63-kb *bla*_OXA-48_-carrying IncL plasmid between *K. pneumoniae* and *E. hormaechei* isolates from human and animal origins [12]. Horizontal transfer of pOXA-48-like plasmids between *K. pneumoniae*, *E coli*, and *E. cloacae* could also occur through co-colonization or co-infection within the same patients [33]. These findings collectively indicate the crucial role of this highly transferable plasmid as the main vehicle for the global dissemination of this carbapenemase gene. The inter-species spread of the *bla*_OXA-48_-48-carrying IncL plasmid among *Enterobacterales* underscores the urgent need for active surveillance of carbapenemase-producing *E. hormaechei*, which could be isolated not only from hospital settings but also from animal reservoirs and environments.

## Conclusion

The high-risk ST78 lineage of *E. hormaechei* has emerged as the primary driver behind the transmission of CPEH in the hospital setting. This lineage has not only acquired various carbapenemase plasmids, such as a *bla*_KPC-2_-carrying pIncFII plasmid and a *bla*_IMP-8_-carrying pIncHI2 plasmid but has also facilitated the transfer of pOXA48-CREH to other lineages, including ST66 and ST90. Our findings highlight the urgent need for genomic surveillance and targeted interventions to control the spread and evolution of this high-risk lineage.

## Supplementary Material

Supplementary materials.pdf

## Data Availability

The genome assemblies of *E. hormaechei* strains in this study are publicly available in GenBank under BioProject PRJNA791797. This article includes all data generated or analyzed during this study. The corresponding authors will make any additional information available upon reasonable request.
