# Supplementary material for: Emergence and transmission of the high-risk ST78 clone of OXA-48-producing Enterobacter hormaechei in a single hospital in Taiwan
Source: Emerg Microbes Infect. 2024 Sep 11;13(1):2404165. doi: 10.1080/22221751.2024.2404165 (PMC11421146; doi:10.1080/22221751.2024.2404165)
Supplement: Supplementary materials.pdf [file TEMI_A_2404165_SM7774.pdf]

Table S1. Primers used in this study.

Target gene		Sequence (5′–3′)	Annealing	Size
IMP	F	GGAATAGAGTGGCTTAAYTC	52°C	232 bp
	R	TCGGTTTAAYAAAACAACCACC		
OXA-48	F	GCGTGGTTAAGGATGAACAC	52°C	438 bp
	R	CATCAAGTTCAACCCAACCG		
KPC	Fm	CGTCTAGTTCTGCTGTCTTG	52°C	798 bp
	Rm	CTTGTCATCCTTGTTAGGCG		
NDM	F	GGTTTGGCGATCTGGTTTTC	52°C	621 bp
	R	CGGAATGGCTCATCACGATC		
VIM	F	GATGGTGTTTGGTCGCATA	52°C	390 bp
	R	CGAATGCGCAGCACCAG		
MCR-9	F	TTCCCTTTGTTCTGGTTG	60°C	1011 bp
	R	GCAGGTAATAAGTCGGTC		
ACT	F	TTGCTCGCGGTGAAATTTCG	56°C	449 bp
	R	CACGTTGGTTTTCACGCCAT		
IncL	F	AACCCGGAGTTCCAGAGAGA	56°C	391 bp
	R	GACCGGCCAAAGTTTCCAAC		
IncHI2	F	ATGAAGCATAACCATTGACATCTG	56°C	252 bp
	R	TCGTAACGGGGTGACAATGG		

(a) 18CRE28 (ST78) Chromosome

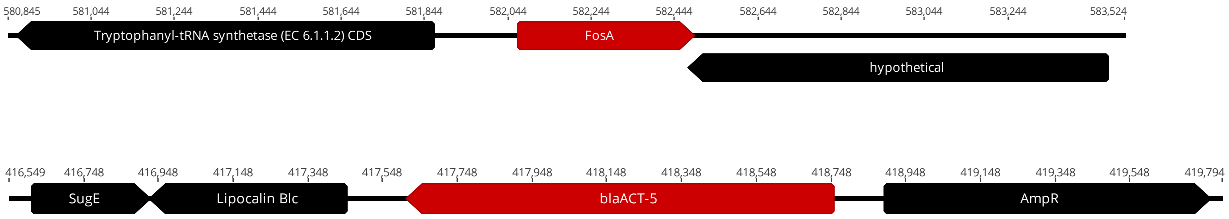

(b) Chromosome-borne AmpC beta-lactamase ACT alignment

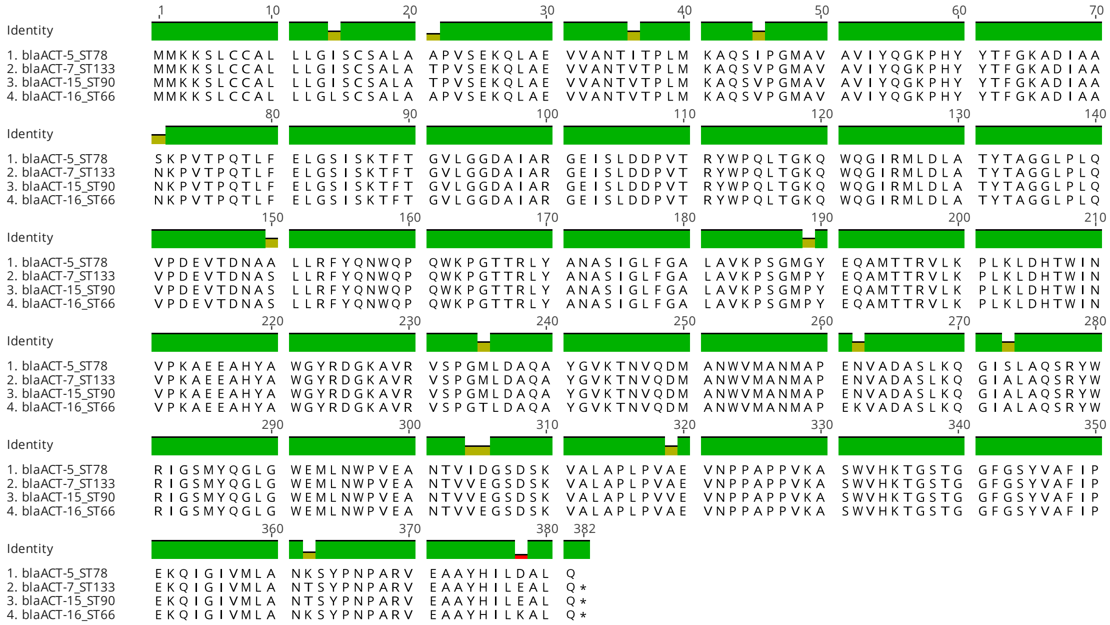

Figure S1. Intrinsic carriage of *bla*$_{ACT}$ gene in different *E. hormaechei* sequence types (STs). (a) Location of *bla*$_{ACT-5}$ gene on the chromosome of an ST78 *E. hormaechei* strain (18CRE28). **(b)** Pairwise alignment of the amino acid sequences of ACT-5, ACT-7, ACT-15, and ACT-16, found in ST78, ST133, ST90, and ST66 *E. hormaechei*, respectively.

(a) BRIG

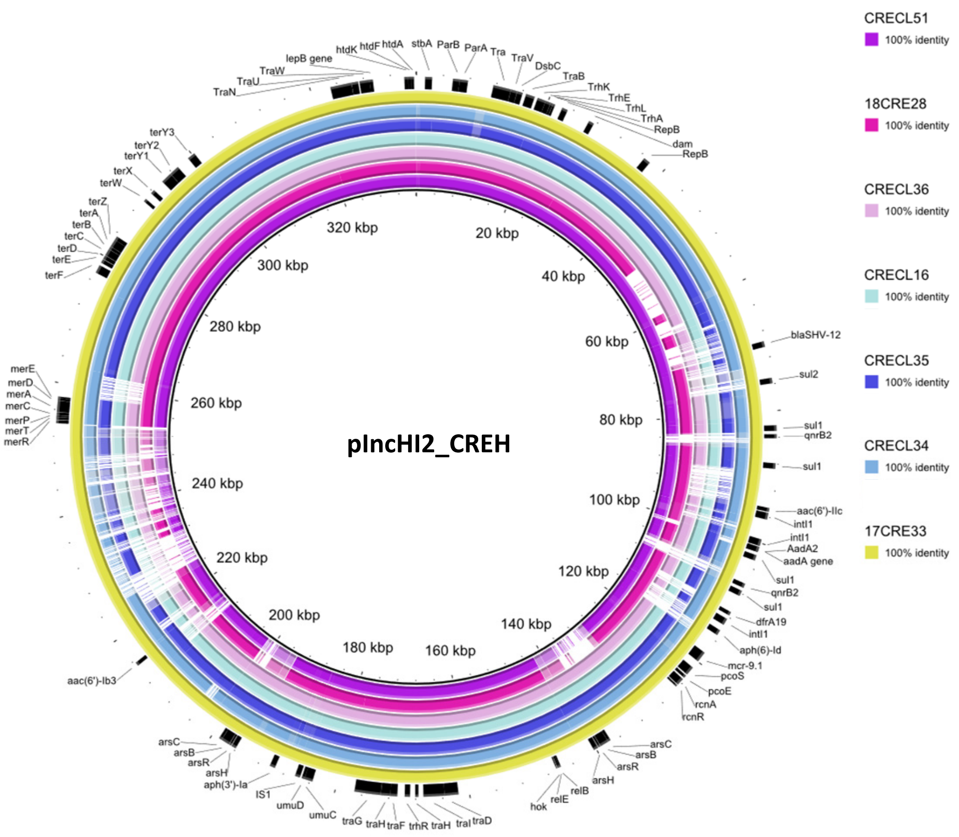

(b) Mauve alignment against pEC-IMP

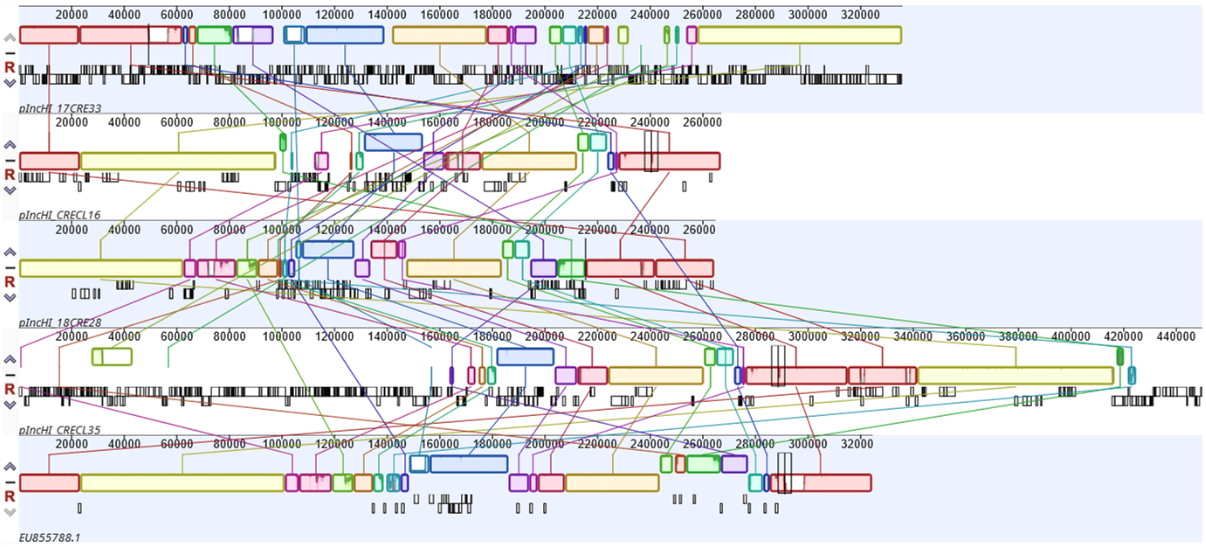

Figure S2. Comparison of pIncHI2 plasmids in representative *E. hormaechei* strains. (a) BRIG comparison of pIncHI2 plasmids, aligned against pIncHI2-17CRE33. **(b)** Mauve alignment of pIncH2 plasmids identified in each of representative strains with pEC-IMPQ (EU855788.1).

(a) Comparison of the conjugal transfer region of pOXA48-CREH

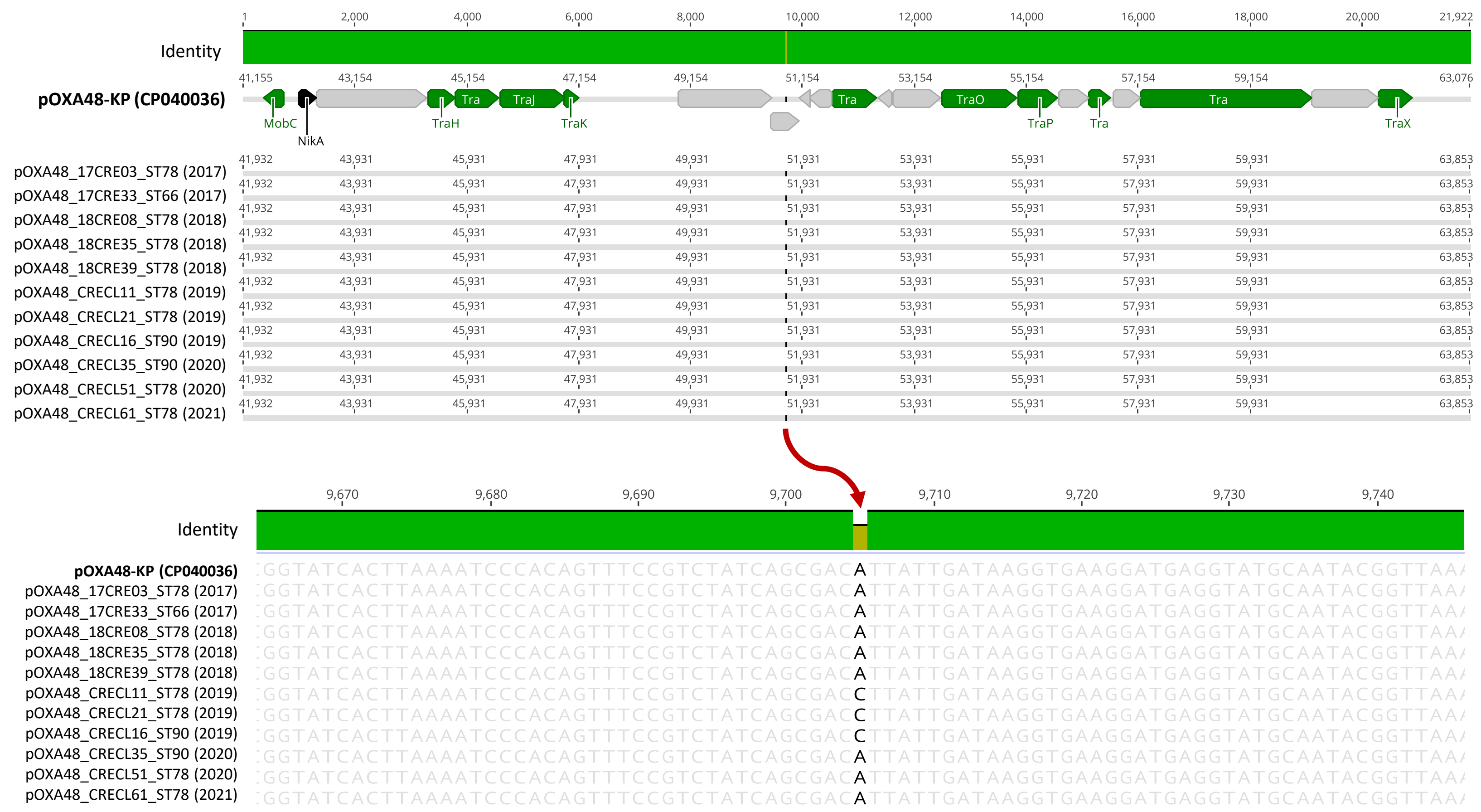

(b) Comparison of the conjugal transfer region on pIncHI2-CREH

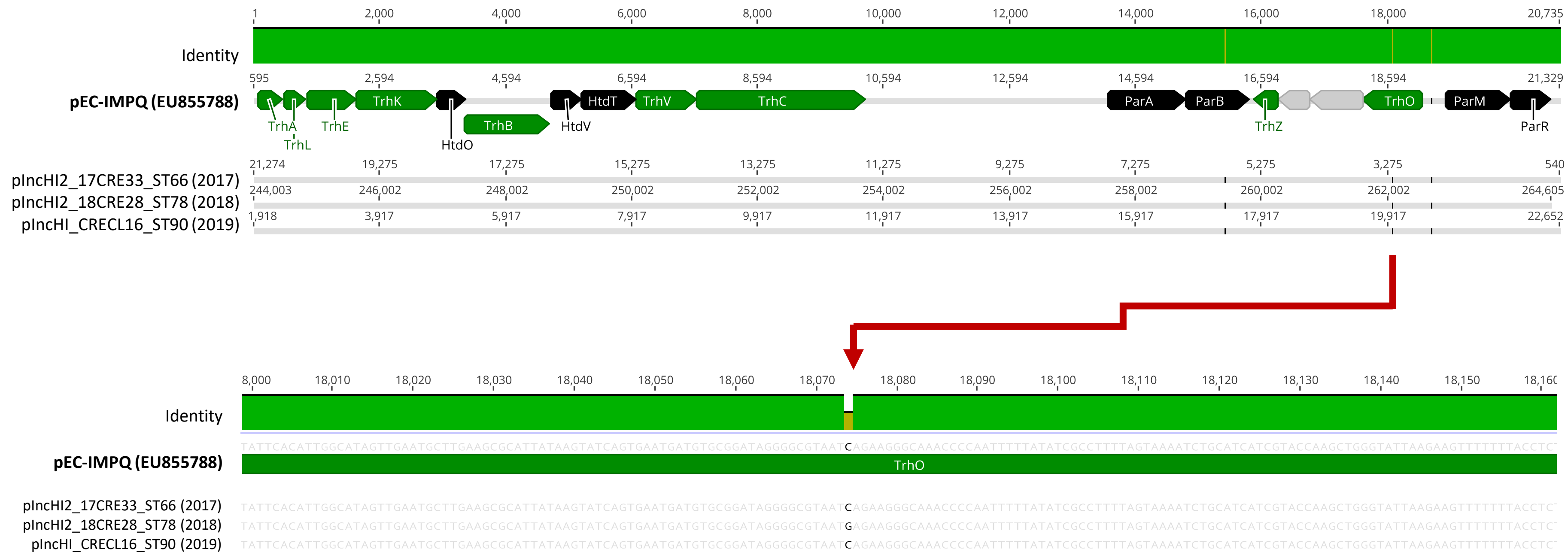

Figure S3. Pairwise alignment of the conjugal transfer region on pOXA48 (a) and pIncHI2 (b) plasmids. (a) Comparison of the conjugal transfer region of pOXA48-CREH plasmids identified in each of the representative strains (n=11) with pOXA48-KP (CP040036), which was identified in a ST11_KL64 *K. pneumoniae* strain**.** An A-to-C transversion occurred in the intergenic region between two hypothetical genes in pOXA48 plasmids in three CPEH strains isolated in 2019. **(b)** Comparison of the conjugal transfer region of representative pIncHI2-CREH plasmids with pEC-IMPQ (EU855788.1). A silent mutation (C-to-G) was detected in the coding region of *trhO* gene of the pIncHI2 plasmid in a ST78 *E. hormaechei* strain isolated in 2018. Conjugal transfer genes are depicted in green.
